# NLRP3 inflammasome activity and periodontal disease pathogenesis–A bidirectional relationship

**DOI:** 10.1111/odi.15005

**Published:** 2024-05-30

**Authors:** Andreea C. Didilescu, Sreedevi Chinthamani, Frank A. Scannapieco, Ashu Sharma

**Affiliations:** 1 Department of Oral Biology, School of Dental Medicine, University at Buffalo, State University of New York, Buffalo, New York, USA; 2 Department of Embryology, Faculty of Dentistry, Carol Davila University of Medicine and Pharmacy, Bucharest, Romania

**Keywords:** bacteria, inflammasome, lipopolysaccharide, macrophage, periodontal disease

## Abstract

**Objective:**

Periodontitis is an inflammatory oral disease that occurs as a result of the damaging effects of the immune response against the subgingival microflora. Among the mechanisms involved, the nucleotide-binding oligomerization domain, leucine-rich repeat-containing proteins family member NLRP3 (NLR family pyrin domain-containing 3), proposed as the key regulator of macrophage-induced inflammation, is strongly associated with periodontal disease due to the bacterial activators. This paper aimed to present key general concepts of NLRP3 inflammasome activation and regulation in periodontal disease.

**Method:**

A narrative review was conducted in order to depict the current knowledge on the relationship between NLRP3 inflammasome activity and periodontal disease. In vitro and in situ studies were retrieved and commented based on their relevance in the field.

**Results:**

The NLRP3 inflammasome activity stimulated by periodontal microbiota drive periodontal disease pathogenesis and progression. This occurs through the release of proinflammatory cytokines IL-1β, IL-18, and DAMPs (damage-associated molecular pattern molecules) following inflammasome activation. Moreover, the tissue expression of NLRP3 is dysregulated by oral microbiota, further exacerbating periodontal inflammation.

**Conclusion:**

The review provides new insights into the relationship between the NLRP3 inflammasome activity and periodontal disease pathogenesis, highlighting the roles and regulatory mechanism of inflammatory molecules involved in the disease process.

## 1 | INTRODUCTION

Periodontitis is an inflammatory oral disease that arises from the damaging effects of the host response against the subgingival microbial community (Hajishengallis et al., 2023; Lamont et al., 2023). If untreated, periodontitis can often lead to tooth loss and has been reported to increase the risk for many systemic diseases such as cardiovascular diseases, diabetes, respiratory infections, and adverse pregnancy and birth-related outcomes. The economic burden worldwide for the control and treatment of periodontitis is approximately $154B loss in the United States and €158B in Europe in 2018 (Botelho et al., 2022). Bacteria alone are insufficient and the tissue damage characteristic of periodontal disease pathology occurs from the self-damaging effects of the immune response elicited to subgingival bacteria. Multiple inflammatory pathways stimulated by the dysbiotic community are involved in driving inflammation associated with periodontitis. A widely accepted model of the pathogenesis of periodontal disease requires subgingival bacterial species, including *Porphyromonas gingivalis*, *Tannerella forsythia*, *Fusobacterium nucleatum, Aggregatibacter actinomycetemcomitans, Treponema denticola*, and *Filifactor alocis*. In this regard, keystone-pathogen hypothesis argues that microbes such as *P. gingivalis* contribute to the disease process by driving a shift toward a dysbiotic microbial community (Hajishengallis et al., 2012). In addition, the environmental factors may play an important role in the onset/progression of the disease (Perez-Chaparro et al., 2014). Microbes release bioactive components such as lipopolysaccharides (LPS), glyco/lipoproteins, proteases, fimbriae, toxins, and genomic DNA. These components induce release of cytokines (de Andrade et al., 2019; Hayward et al., 2018) that activate leukocytes, the complement system, antibodies and autoantibodies, inflammatory cytokines and reactive oxygen species to drive this chronic inflammatory process (Hajishengallis, 2010; Hajishengallis & Chavakis, 2021; Liu et al., 2017).

Among the inflammatory cascades activated, the role of inflammasome triggered inflammatory pathways has been recognized in recent years in the pathogenesis of periodontitis. Inflammasomes, first described by Martinon et al. (2002), are multiprotein cytosolic complexes that are assembled in response to an infection or cellular stress. Their activation results in the release of inflammatory cytokines, mainly IL-1β, IL-18, and lytic cell death of the infected host cell. Typical, or canonical, inflammasome activation depends on assembly of a multiprotein complex that senses danger signals and recruits pro-caspase-1 via ASC protein (the adaptor molecule apoptosis-associated speck-like protein containing a caspase recruitment domain, CARD) to cause the maturation of pro-IL-1β and pro-IL-18 into mature IL-1β and IL-18 and their release (Broz & Dixit, 2016). Non-canonical inflammasome activation, on the other hand, involves direct sensing of pathogen-associated molecules by inflammatory caspases-4/−5 in humans and caspase-11 in mice. Upon activation, inflammasomes also promote an inflammatory and lytic form of cell death, called pyroptosis, caused by plasma pore-formation by the gasdermin D (GSDMD) N-terminal domain released by the caspase-mediated processing of the full-length GSDMD (Kayagaki et al., 2015; Shi et al., 2015). Dysregulated inflammasome activity is a hallmark of many multi-factorial diseases with an inflammatory component, such as autoimmune disorders; cardiovascular diseases; bacterial, fungal, or viral infections; and cancer and neurological diseases (Man, 2018; Strowig et al., 2012; Walsh et al., 2014). The contribution of inflammasomes in periodontitis pathogenesis is supported by studies that show that increasingly higher levels of inflammasome components are found in the gingival tissues from patients with chronic periodontitis than from healthy controls (Park et al., 2014). The notion that the inflammasome might be a crucial unit of the host immunity in periodontitis is also supported by studies showing that gingival epithelial cells, which are important parts of the immune response to periodontal bacteria, express a functional inflammasome and release cytokines and chemokines as important contributors to further development of periodontal disease (Jayaprakash Demirel et al., 2023; Yilmaz et al., 2010).

To date, NLRP3, the best studied inflammasome, has been shown to be elevated and primed in the presence of periodontal inflammation, playing an important role in host inflammatory response (Marchesan et al., 2020). Since it is not possible to cover the field of rapidly growing inflammasome biology in a single review, the objective of this paper was to summarize key general concepts about NLRP3 inflammasome activation and regulation, and importantly, what is known so far of their role in periodontal disease.

## 2 | NLRP3 INFL AMMASOME ACTIVATION

Inflammasomes are intracellular multimeric protein complexes that respond to various pathogen derived pathogen-associated molecular patterns (PAMPs) and host derived danger-associated molecular patterns (DAMPs). Inflammasomes are defined by their sensor protein, which oligomerizes to form a pro-caspase-1 activating platform in response to DAMPs or PAMPs. There are five main members that have been confirmed to form inflammasomes: the nucleotide-binding oligomerization domain (NOD), leucine-rich repeat (LRR)-containing proteins (NLR) family members NLRP1 (NLR family pyrin domain-containing 1), NLRP3 (NLR family pyrin domain-containing 3, also known as NALP3) and NLRC4 (NLR family CARD domain-containing 4, also known as IPAF), as well as absent-in-melanoma 2 (AIM2) and pyrin (Lamkanfi & Dixit, 2014; Sharma & Kanneganti, 2016). In addition, other members that include NLRP2, NLRP6, NLRP7, NLRP12, and IFI16 were also reported to form inflammasomes (Davis et al., 2011; Ranson et al., 2017). In the case of NLRP1, NLRP3, AIM2, and pyrin, an adaptor protein known as apoptosis-associated speck-like protein containing a caspase-recruitment domain (ASC) facilitates the recruitment of pro-caspase-1 to the inflammasome complex. Different inflammasomes are activated by different stimuli (Zheng et al., 2020). For example, NLRP1 usually requires MDP/NOD2 for activation (Pedra et al., 2009). NLRC4 can be activated by cytosolic flagellin in cells infected with Salmonella, Legionella, and Pseudomonas spp (Gram et al., 2021; Pereira et al., 2011; Sutterwala et al., 2007). NLRP3 inflammasome components are activated by a variety of stimuli, including microbial products and endogenous signals, such as urate crystal, silica, amyloid fibrils, and ATP (Kelley et al., 2019).

The NLRP3 inflammasome has been the major target of studies focusing on the role of macrophages in many inflammatory diseases including periodontitis. It has been proposed as the key regulator of macrophage-induced inflammation (Kelley et al., 2019; Zhang et al., 2020). However, in addition to macrophages, inflammasomes can be formed in many cell types including monocytes, dendritic cells, neutrophils, and epithelial cells. The proposed model for activation of NLRP3 inflammasome requires two independent signals: a priming signaling (signal I) often induced by PAMPs, such as LPS, DAMPs, and proinflammatory cytokines to cause NF-κB-mediated induction of the inflammasome protein, pro-caspase, pro-IL-1β, and pro-IL-18 expression (Bauernfeind et al., 2009), and a second activation signal (signal II) that induces assembly of the inflammasome complex and the recruitment and activation of caspases (He et al., 2016). NLRP3 assembly can occur by a large number of stimuli (signal II) including both DAMPs and PAMPs; DAMPs include monosodium urate crystals, calcium influx, mitochondrial reactive oxygen species, and extracellular ATP, while PAMPs derive from viral and bacterial sources such as toxins (nigericin), LPS, lipoproteins, RNA, and DNA.

### 2.1 | The canonical and non-canonical pathways of inflammasome activation

**FIGURE 1:**
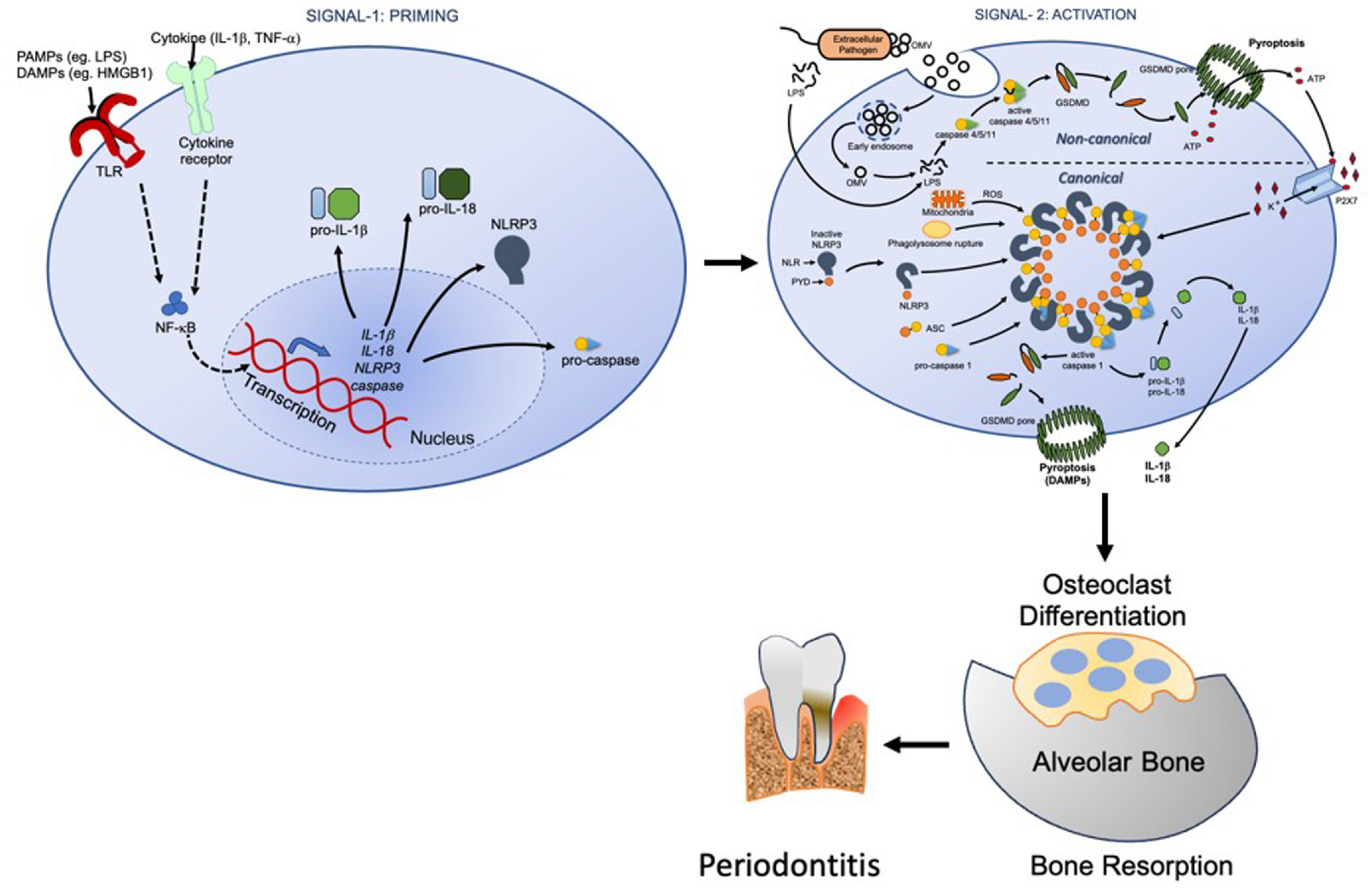
Schematic overview of the NLRP3 inflammasome complex formation and signaling, by two activation pathways: canonical and non-canonical. GSDMD, gasdermin D; LPS, lipopolysaccharide; OMV, outer membrane vesicle; ROS, reactive oxygen species.

NLRP3 inflammasome assembly and activation can occur by two different pathways: a canonical pathway and a non-canonical pathway. Caspase-1 in the canonical inflammasome, and caspase-4 (caspase-11 in mice) and caspase-5 in the non-canonical, are the central effector enzymes activated upon stimulation (Broz & Dixit, 2016). The consequences of canonical and non-canonical inflammasome activation of NLRP3 are quite similar, and the non-canonical pathway can ultimately converge and activate the canonical pathway (Figure 1).

In a typical canonical activation of NLRP3 inflammasome, in response to stimuli, active caspase-1 is formed following the assembly of a multiprotein complex consisting of NLRP3 protein, the adaptor protein ASC, and pro-caspase-1. In the assembly of this multiprotein complex, ASC links the NLR receptor to procaspase-1 using its PYD to interact with the PYD of the NLR and its CARD to interact with the CARD of procaspase-1 (Pedra et al., 2009). The central effector protein is caspase-1 (cysteine protease) that, after activation, cleaves cytosolic pro-IL-1β and pro-IL-18 to their active forms IL-1β and IL-18, which are then secreted by the cells (Pedra et al., 2009).

In the case of canonical activation, NLRP3 inflammasome can be recruited in response to various PAMPs including LPS, extracellular ATP, ion flux, lysosome damage, and production of reactive oxygen species induced by mitochondrial dysfunction. This then leads to NLRP3 dependent caspase-1 activation (Sutterwala et al., 2006). In this situation, TLR stimulation promotes extracellular release of ATP that in turn stimulates the purinergic receptor P2X7 to cause potassium efflux, resulting in NLRP3 inflammasome activation. In the case of non-canonical inflammasome activation, LPS internalization into the cytosol via bacterial infection or transfection causes caspase-4/5/11 activation independently of TLR. In this scenario, aggregated LPS molecules in the cytoplasm directly bind and activate caspase 4/5 or 11 (Kayagaki et al., 2011; Kelley et al., 2019). This ultimately leads to caspase-4/5/11 dependent pyroptosis through cleavage of the pore-forming protein GSDMD. The pyroptosis and the subsequent release of ATP can then trigger a secondary activation of the canonical NLRP3 and caspase-1 activation (Devant & Kagan, 2023). Thus, the non-canonical pathway is indirectly linked to the canonical pathway (Kayagaki et al., 2013). In contrast to canonical inflammasome activation that depends on the formation of multiprotein scaffold complex, non-canonical inflammasome activation requires direct binding of intracellular LPS to caspases. It is proposed that positively charged motifs in the CARD domain of caspase-4/5 and caspase-11 are involved in LPS binding via negatively charged phosphates of the lipid A backbone in LPS. Binding of caspase-11 to LPS triggers its oligomerization and activation (Shi et al., 2014). The intracellular LPS sensing is attributed to CD14-mediated cytosolic localization of LPS in a TLR4-independent manner (Vasudevan et al., 2022). It is observed that internalization of LPS is enhanced in the presence of LPS-binding protein (LBP), which promotes LPS aggregation without affecting the LPS stimulatory potency (Kitchens & Munford, 1998). In humans, a plethora of proteins are expressed in response to activation of the non-canonical pathway after *Escherichia coli* LPS recognition in macrophages (Lorey et al., 2017). Beside the above-mentioned pathways, an alternative pathway has been described in human monocytes. It is reported that caspase-4 and −5 mediate IL-1α and IL-1β release from human monocytes after LPS stimulation via a non-canonical one-step pathway of inflammasome (Vigano et al., 2015). This one-step pathway requires Syk activity and Ca^2+^ flux initiated by CD14/TLR4-mediated LPS internalization. In this process, caspase-4 remains uncleaved but undergoes a rapid processing, whereas caspase-5 is cleaved upon LPS-stimulation (Gaidt et al., 2016; Kelley et al., 2019).

To date, the role of LPS in periodontal inflammation has been widely explored in the context of TLR mediated inflammatory pathways. Nevertheless, how the pathogenic potential and severity of periodontitis varies depending on the heterogeneity of the LPS from oral bacteria remains to be determined (Dixon & Darveau, 2005; Sedghi et al., 2021). It has been suggested that lipid A modifications in pathogenic bacteria combat caspase-11-mediated surveillance mechanisms (Kayagaki et al., 2013).

It is thought that the major role of the non-canonical inflammasome is to promote the eradication of gram-negative bacteria that escape killing by phagosomes after invading the cytoplasm. The non-canonical inflammasome activation helps in the removal of infected cells by pyroptosis. Non-canonical inflammasome activation also alerts neighboring cells through the release of alarmins and DAMPs, leading to the release of cytokines via the activation of canonical NLRP3 inflammasome in caspase-1 dependent pathway.

## 3 | PERIODONTAL PATHOGEN LIGANDS IN NLRP3 ACTIVATION

### 3.1 | Modulation of NLRP3 activity by gram-negative bacteria

A well-studied gram-negative subgingival organism thought to be involved in driving periodontitis, *P. gingivalis*, was shown to induce gene expression of IL-1β and IL-18 cytokines in gingival tissues and through NLRP3 inflammasome activation to induce alveolar bone loss in mice (Yamaguchi et al., 2017). Later, studies showed that *P. gingivalis* can induce NLRP3 activation through both canonical and non-canonical pathways (Ding et al., 2020; Okano et al., 2018).

Although what *P. gingivalis* factors at the molecular level activate inflammasome awaits further research, one possible candidate may be outer membrane vesicles (OMVs). OMVs are 50–250 nm-diameter spherical, bilayered, membranous structures that contain LPS, outer membrane proteins and other constituents, which are released into the external environment from the outer membrane of the gram-negative bacteria (Beveridge, 1999). Indeed, OMVs released from *P. gingivalis* during growth and/or stress have been shown to cause inflammasome activation and pyroptosis in macrophages (Fleetwood et al., 2017). The main component in *P. gingivalis* OMVs which is likely to be responsible for inflammasome activation might be LPS. In fact one study showed *P. gingivalis* LPS and a hypoxic environment synergistically induced NLRP3 inflammasome in human gingival fibroblasts, leading to high levels of interleukin-1β and GSDMD-mediated pyroptosis response (Yang et al., 2021). Another study indicated that NLRP3 activation depends on *P. gingivalis* LPS or pretreatment with ATP, and not on *P. gingivalis* infection (Guo et al., 2015). It remains to be seen how *P. gingivalis* LPS enters the host cell to induce non-canonical and canonical pathways of inflammasome activation, but one possibility is that LPS is delivered into the cytoplasm following endocytosis of OMVs. It should be noted that *P. gingivalis* expresses two distinct LPS macromolecules containing different glycan repeating units: an O-LPS with O-antigen tetrasaccharide repeating units, and an A-LPS with phosphorylated branched mannan repeating units (Rangarajan et al., 2008). Moreover, nonphosphorylated penta-acylated and nonphosphorylated tetra-acylated species are present in lipid A from total LPS and in lipid A from A-LPS. To what extent these unique attributes of *P. gingivalis* LPS might be responsible for inflammasome regulation (enhance or dampen) remains to be determined.

*F. nucleatum* is another important organism of the subgingival community associated with periodontitis. It is considered a key pathogen in periodontal disease. It bridges early commensal and later colonizing pathogens, contributing to dental plaque formation (Kolenbrander et al., 2010). *F. nucleatum* infection of gingival epithelial cells has been shown to cause NLRP3 inflammasome-dependent secretion of IL-1β and danger signals ASC and HMGB1 (Bui et al., 2016). Intriguingly, *P. gingivalis* has been shown to suppress inflammasome induction by *F. nucleatum*. *P. gingivalis* is able to suppress the secretion of IL-1β induced by *F. nucleatum* by blocking the *F. nucleatum*-mediated activation of caspase-1 (Taxman et al., 2012). It is proposed that suppression of innate immune response by *P. gingivalis* could facilitate the maintenance of the chronic state of infection during periodontal diseases.

During periodontitis development, the number of inflammatory M1 (classically activated) macrophages in the oral cavity has been found to increase significantly (Gonzalez et al., 2015; Yu et al., 2016). A number of periodontal bacteria and their components including *P. gingivalis* and *F. nucleatum* OMVs are implicated in M1 macrophage development (Champaiboon et al., 2014; Chen et al., 2022). Evidence suggests that NLRP3 inflammasome activation leads to M1 macrophage polarization in inflammatory root resorption and periodontitis (Han et al., 2022; Hou et al., 2022; Zhang et al., 2020). Taken together, these findings suggest that M1 polarization observed in periodontitis leading to increased alveolar bone loss may occur due to heightened inflammasome activation.

Intriguingly, NLRP3 and AIM2 inflammasomes and their downstream IL-1 targets have been shown to be differentially regulated in gingival fibroblasts (GFs) by supragingival and subgingival biofilms (Bostanci et al., 2011). *F. nucleatum* and *P. gingivalis* present in subgingival biofilms have been shown to dysregulate inflammasome expression in GFs. Exposure of GFs to *F. nucleatum* in the presence of ATP increases IL-1β production and upregulates NLRP3 and AIM2 expression. On the other hand, *P. gingivalis* in the presence of ATP increases IL-1β expression and downregulates NLRP3 expression without any effect on AIM2 expression (Aral et al., 2022). Moreover, in vitro studies have shown that GFs challenged with subgingival biofilms promoting periodontitis at lower levels display increased gene expression of NLRP3, caspase-1, ASC, AIM2, IL-1β, and IL-18, whereas GFs display decreased gene expression of these factors when challenged with higher levels of subgingival biofilm (Bostanci et al., 2011). The authors of these studies suggested that decreased transcription of inflammasome machinery in response to subgingival biofilms, leading dampening of host immune responses, might allow pathogen survival and persistence.

### 3.2 | Modulation of inflammasome activity by gram-positive bacteria

The inflammasome activation by gram-positive oral streptococci is worth noting. While streptococci are considered to be the part of normal oral flora, they also colonize extraoral sites and contribute to systemic diseases (Lockhart et al., 2009). Moreover, recent studies have indicated that periodontitis pathogenesis is exacerbated by the presence of streptococcal species, such as *Streptococcus gordonii*, which can act as accessory periodontal pathogens (Park et al., 2019; Ramsey et al., 2011). Previous studies have shown that many different types of inflammasomes are activated by cell wall components of gram-positive oral streptococci. For example, *S. gordonii* is able to increase inflammatory responses of macrophages via NLRP6 inflammasome via its lipoteichoic acid (LTA) (Hara et al., 2018; Metcalfe et al., 2023). *Streptococcus mutans*, considered a dental caries pathogen, can also induce NLRP3 and NLRC4 activation in infected THP-1 monocytes (Song et al., 2018). Moreover, *Enterococcus faecalis* LTA can activate NLRP3 inflammasome via both the canonical and non-canonical pathways, and this can potentially promote apical periodontitis (Park et al., 2023). Thus, the oral streptococci might contribute to the development of periodontal diseases via the activation of different types of inflammasomes.

## 4 | NLRP3 POLYMORPHISM AND PERIODONTITIS

In addition to the signals provided by a pathogen for NLRP3 activation, the polymorphism related to higher transcriptional activation of the NLRP3 gene should be considered as a risk factor in the pathogenesis of periodontal disease (de Alencar et al., 2020; Isaza-Guzman et al., 2016; Mahendra et al., 2021; Mahmood & Abbas, 2023; Miskiewicz et al., 2015).

Specific genetic polymorphism in NLRP3 (T/C genotype) are associated with periodontal disease susceptibility. Moreover, males carrying this genetic polymorphisms are at a greater susceptibility than women for developing the disease (Honma & Honma, 1988; Isaza-Guzman et al., 2016). The mutated allele C has been shown to be correlated with the higher transcriptional activity of the NLRP3 gene when compared to the wild-type T allele (Hitomi et al., 2009). An overexpression of the NLRP3 inflammasome and a downregulation of NLRP3 inhibitors have been observed in the gingival tissue of patients with periodontal disease (Aral et al., 2020; Garcia-Hernandez et al., 2019; Xue et al., 2015). The NLRP3 overexpression can be caused by signals induced by recognition of PAMPs, such as LPS, and DAMPs, such as ATP released by dying or injury of host cells, by PRRs (Yilmaz & Lee, 2015). To this end, in vitro studies have shown that periodontopathogenic bacteria, such *P. gingivalis*, *A. actinomycetemcomitans*, and *F. nucleatum*, are involved in the increased expression of NLRP3. The increased expression of NLRP3 by these pathogens leads to the maturation and secretion of IL-1β (Belibasakis & Johansson, 2012; Bui et al., 2016; E. Park et al., 2014) and IL-18 (Belibasakis & Johansson, 2012; Bostanci et al., 2009), and the pyroptotic cell death (E. Park et al., 2014) leading to an exacerbated inflammation in the periodontium tissue.

## 5 | SALIVARY, GINGIVAL TISSUE AND CREVICUL AR FLUID NLRP3 LEVELS AS POTENTIAL BIOMARKERS FOR PERIODONTAL DISEASE

Clinical studies have evaluated NLRP3 inflammasome expression in periodontal disease, using tissue, saliva and/or gingival crevicular fluid (GCF) samples. Studies performed on gingival tissue have shown that NLRP3 expression was significantly higher in different types of periodontal diseases as compared to healthy individuals (Aral et al., 2020; Bostanci et al., 2009; Cheng et al., 2017). Several inflammasome components were expressed at higher levels in saliva, GCF, and periodontal tissues (Marchesan et al., 2020). Recent findings have shown that in cases with moderate-to-severe and generalized periodontal breakdown, increased salivary levels of NLRP3, ASC, and IL-1β were recorded, as compared to healthy controls (Isaza-Guzman et al., 2017), suggesting that NLRP3 may represent an independent predictor of disease risk during different stages of periodontal disease. No significant differences between the groups with respect to caspase-1 levels were reported (Isaza-Guzman et al., 2017), a non-canonical pathway being probably activated by gram-negative periodontal pathogens. In another study, salivary levels of NLRP3 were found significantly higher in chronic periodontitis patients as compared to healthy controls (Mitra et al., 2022). Increased salivary levels of NLRP3, ASC, and IL-1β were also observed in systemic healthy patients with periodontitis, and diabetic patients with periodontitis, when compared with healthy periodontal patients (Isola et al., 2022). In the same study, periodontitis was found significant predictor of salivary NLRP3 (Isola et al., 2022). Conversely, periodontal therapy contributed to decreasing of the NLRP3 levels in GCF samples of chronic periodontitis patients (Shahbeik et al., 2021).

In addition, systemic diseases such as diabetes may enhance NLRP3 expression in patients with chronic periodontitis, with an increase in IL-1β production (Garcia-Hernandez et al., 2019). In turn, it has been hypothesized that periodontitis, in patients with type-II DM, may contribute, through its chronic inflammatory effects supported by an abundant oral microflora, to a further upregulation of the salivary and serum NLRP3 concentrations (Isola et al., 2022). In a recent study, patients with chronic hepatitis C and periodontitis were found with significantly more elevated levels of NLRP3, caspase-1, and IL-18 in GCF samples, than systemic healthy patients with periodontitis (Surlin et al., 2021).

### 6 | CONCLUDING REMARKS

Many advances in the field knowledge show that the NLRP3 inflammasome activity stimulated by periodontal microbiota drive periodontal disease pathogenesis and progression. Information acquired on genetic polymorphism and microbiome interactions during periodontal disease onset and progression allows health-care providers to approach periodontal treatment based on the concept of precision medicine. However, there are still unknown answers to several questions, such as how does the LPS of periodontal pathogens act in the non-canonical induction of the inflammasome pathway; which inflammasome is mostly preferred by specific bacterial ligands; and how do the inflammasomes cross-react to periodontal pathogens that are able to activate them simultaneously. Regulation of inflammasome activity and therapeutic interventions targeting structures related to inflammasome signaling constitute promising areas of basic and translational research.

## Data Availability

Data sharing is not applicable to this article as no new data were created or analyzed in this study.
